# Responses of Crops to Abiotic Stress

**DOI:** 10.3390/plants14131927

**Published:** 2025-06-23

**Authors:** Giora Ben-Ari, Georgios Koubouris

**Affiliations:** 1 Institute of Plant Sciences, ARO, The Volcani Institute, Rishon LeZion 7528809, Israel; 2 Hellenic Agricultural Organization (ELGO) DIMITRA, Institute of Olive Tree, Subtropical Crops and Viticulture, 73134 Chania, Greece

## 1. Introduction

During recent years, agriculture has been undergoing a significant transformation in response to contemporary economic pressures and the escalating impact of climate change. Global climate change is recognized as a critical threat to the stability and sustainability of natural ecosystems. It represents a complex and multifactorial process involving shifts in key environmental parameters, including elevated atmospheric CO_2_ concentrations, temperature fluctuations, intensified heat waves, and changes in precipitation patterns. These alterations contribute to the emergence and proliferation of novel pests, weeds, and pathogens, thereby exerting additional stress on agricultural systems.

Climate change exerts profound effects on plant physiology, particularly through abiotic stressors that often interact synergistically to induce morphological, physiological, biochemical, and molecular alterations. These stress-induced changes inhibit plant growth, development, and productivity, ultimately leading to yield reductions. Among the various growth stages, the sexual reproductive phase is especially susceptible to the deleterious effects of abiotic stress, thus severely limiting reproductive success and crop yield. This Special Issue will bring together recent advancements in understanding the impacts of climate change on crop yield and quality.

## 2. Overview of the Published Manuscripts

The responses of crops to abiotic stresses involve many transcription factor families, which induce varied responses in different plants. This Special Issue presents studies of six gene families involved in these responses.

Heat shock factors (*Hsfs*) are a group of transcription factors that regulate the expression of heat shock proteins (HSPs) in response to stress. The regulatory capacity of *Hsfs* is largely due to their ability to preserve protein stability under stressful conditions. The first study presented in this Special Issue found 22 Hsf genes in the tea plant *Camellia sinensis*. These genes were classified as belonging in three major subfamilies: CsHsfA, CsHsfB, and CsHsfC. The expression of many *CsHsf* genes was found to be affected by abiotic stresses such as low temperature and excess light. These results lay a solid groundwork for further investigations into *CsHsf* involvement in the responses of the tea plant *Camellia sinensis* to abiotic stresses [1]. 

In response to abiotic stresses, plants produce heat shock proteins (Hsps), which are essential for cell survival during periods of stress. Hsps are classified as Hsp100, Hsp90, Hsp70, Hsp60, Hsp40, and Hsp20 based on their molecular weight. Hsp40 proteins are the most prevalent in eukaryotic organisms. *ZmDnaJ* (HSP40s) genes from maize were analyzed and classified into three types. Analysis of cis-regulatory elements in *ZmDnaJ* promoters suggested their involvement in stress responses, growth and development, and phytohormone sensitivity in maize. RNA-seq analysis showed the constitutive expression of most *ZmDnaJ* genes, some specifically in pollen and endosperm. Various genes of this gene family responded to salinity, heat, and cold stresses, indicating potential interaction between stress regulatory networks [2].

Another gene family that was investigated in the tea plant is the *BES1* gene family. A total of 10 *BES1* genes were identified in the tea genome. Analysis of the promoter regions in these genes revealed two types of light-responsive cis-elements. An expression analysis showed that some of these genes are significantly upregulated under light exposure. The study has provided insights into the functional roles of the BES1 gene family in response to abiotic stresses such as exposure to high-intensity light [3].

The depletion of the ozone layer has resulted in elevated ultraviolet-B (UV-B) radiation levels, posing a significant risk to terrestrial plant growth. The responses of WRKY transcription factors in *Rhododendron chrysanthum* Pall. (*R. chrysanthum*) to UV-B stress and their regulation of flavonoid synthesis were studied. The study revealed changes in the expression of 113 flavonoid-related metabolites and 42 associated genes, with WRKY transcription factors showing significant correlation with these alterations. It should be noted that WRKY transcription factors can influence the expression of key enzyme genes in the flavonoid metabolic pathway, thereby affecting metabolite production [4].

Aldehyde dehydrogenase (ALDH) is effective at eliminating active aldehyde molecules in plants. The study presented in this Special Issue explores the impact of the ALDH gene family on melon growth, their development, and their expression patterns in various tissues and under different stress conditions. The expression patterns of various genes in this family were regulated under all six tested biotic and abiotic stress parameters, namely salinity, frost, waterlogging, powdery mildew, *Fusarium* wilt, and gummy stem blight. The study paves the way for future genetic improvements in melon molecular breeding [5].

An investigation of the MYB transcription factors in *Paulownia fortunei*, used cloning of the *PfMYB44* gene from *Paulownia fortunei*. Overexpression-*PfMYB44* plants were constructed, and physiological and molecular analysis showed that *PfMYB44* could positively regulate salt and drought stresses in Arabidopsis. Under conditions of drought stress, AtP5CS, AtCAT1, AtNCED3 and AtSnRK2.4 expression levels in transgenic lines were also significantly induced. Salt stress induced the expression of *AtNHX1*, *AtSOS1*, *AtSOS2* and *AtSOS3* genes. In conclusion, the functional study of *PfMYB44* laid the foundation for the study of *Paulownia* stress resistance and delved into its stress resistance mechanism and the cultivation of new stress-resistant varieties [6].

One of the plant hormones involved in the responses of plants to abiotic stress is abscisic acid (ABA). Two studies published in this Special Issue explore the role of the ABA plant hormone in the responses of rice and *R. chrysanthum*. 

Abscisic acid (ABA) influences *R. chrysanthum*’s metabolic responses under UV-B stress. It was observed that UV-B stress negatively impacts the plant’s photosynthetic machinery, disrupting multiple metabolic processes. Multi-omics analysis revealed that applying ABA mitigates the detrimental effects of UV-B on photosynthesis and bolsters the plant’s antioxidant defenses. These findings underscore ABA’s crucial function in improving plant resistance to UV-B stress and offer novel insights into plant stress biology [7].

In rice, *OsNCED3*-overexpressing lines have increased ABA content by up to 50.90% and improved the transcription levels of numerous genes involved in stress responses, thus significantly enhancing seedling survival rates. They also increased the dry weight contents of the total chlorophyll, proline, soluble sugar, and starch, in addition to increasing the activity of antioxidant enzymes of rice seedlings and reducing the contents of O_2_^−^, H_2_O_2_, and malondialdehyde under hydroponic alkaline stress conditions. The results of these findings suggest that inducing *OsNCED3* upregulates endogenous ABA levels and the expression of additional stress response genes. This represents an innovative molecular approach for enhancing the alkaline tolerance of rice seedlings [8].

Other interesting studies published in this Special Issue investigate various aspects of plant responses to abiotic stress.

One study examined the effects of heat priming and heat stress on delayed germination, shoot length, and shoot fresh and dry weight under elevated-temperature conditions. The results demonstrate that while heat stress delayed germination in progeny, heat priming significantly accelerated germination rates. Furthermore, heat priming helped to maintain low levels of reactive oxygen species (ROS) and malondialdehyde (MDA), contributing to greater biomass accumulation. These findings suggest that heat priming enhances heat tolerance in rice [9].

*Sideritis cypria* Post is a promising medicinal and aromatic plant. Another study published in this Special Issue described the cultivation of *S. cypria* plants hydroponically, using nutrient solutions (NSs) with different N and Cu levels, combined with the foliar spraying of Zn. Excess Cu increased lipid peroxidation (MDA) at low and moderate N levels in the NS, while foliar Zn reduced both MDA and hydrogen peroxide contents, contingent upon Cu and N levels. These results may be utilized to aid in optimizing nutrient management strategies for cultivating *S. cypria*, considering the potential benefits of Zn foliar applications under conditions of Cu contamination [10]. 

The cold stress response of the *D. officinale* plant is the subject of another article that we have included. The results suggest that the glycine metabolism-related genes *Dca003913* and *Dca022726* play pivotal roles in both cold and drought stress adaptation. Carbohydrate metabolism showed specific changes in reaction to freezing conditions. These involved a variety of hormonal responses. The study highlights the roles of metabolic reprogramming and RNA splicing in responses to cold [11]. 

The final study evaluated the performance of two avocado rootstocks under four conditions of water stress during the nursery stage. Plant height, leaf area (LA), dry matter (DM), and carbon (OC) contents in the roots, stems, and leaves were measured. One rootstock of the four measured aspects was found to have higher sensitivity to extreme changes in water availability. These insights are crucial for selecting rootstocks that ensure optimal performance under varying conditions of water availability, enhancing productivity and sustainability [12].

## 3. Concluding Remarks

The manuscripts featured in this Special Issue encompass studies covering varied plant responses to abiotic stress. As Guest Editors of this Special Issue, titled “Responses of Crops to Abiotic Stress”, we would like to thank all the authors for submitting such interesting manuscripts. The studies reported in this collection will help farmers to mitigate the negative effects of abiotic stresses that have increased dramatically in recent years, which are likely to increase in intensity in the future. It has been a pleasure to read and learn from their works. We would also like to thank the reviewers for their valuable comments on the manuscripts, as well as the Editorial Office for their support.
